# Updated and Validated Pan-Coronavirus PCR Assay to Detect All Coronavirus Genera

**DOI:** 10.3390/v13040599

**Published:** 2021-04-01

**Authors:** Myndi G. Holbrook, Simon J. Anthony, Isamara Navarrete-Macias, Theo Bestebroer, Vincent J. Munster, Neeltje van Doremalen

**Affiliations:** 1 Laboratory of Virology, National Institute of Allergy and Infectious Diseases, National Institutes of Health, Hamilton, MT 59840, USA; myndi.holbrook@nih.gov (M.G.H.); vincent.munster@nih.gov (V.J.M.); 2 Department of Pathology, Microbiology, & Immunology, School of Veterinary Medicine, University of California, Davis, Davis, CA 95616, USA; sjanthony@ucdavis.edu (S.J.A.); i.navarrete.macias@gmail.com (I.N.-M.); 3 Department of Viroscience, Erasmus MC Rotterdam, 3015 GE Rotterdam, The Netherlands; t.bestebroer@erasmusmc.nl

**Keywords:** coronavirus, PCR, pan-CoV PCR, detection, pandemic, reservoir host

## Abstract

Coronavirus (CoV) spillover events from wildlife reservoirs can result in mild to severe human respiratory illness. These spillover events underlie the importance of detecting known and novel CoVs circulating in reservoir host species and determining CoV prevalence and distribution, allowing improved prediction of spillover events or where a human–reservoir interface should be closely monitored. To increase the likelihood of detecting all circulating genera and strains, we have modified primers published by Watanabe et al. in 2010 to generate a semi-nested pan-CoV PCR assay. Representatives from the four coronavirus genera (α-CoVs, β-CoVs, γ-CoVs and δ-CoVs) were tested and all of the in-house CoVs were detected using this assay. After comparing both assays, we found that the updated assay reliably detected viruses in all genera of CoVs with high sensitivity, whereas the sensitivity of the original assay was lower. Our updated PCR assay is an important tool to detect, monitor and track CoVs to enhance viral surveillance in reservoir hosts.

## 1. Introduction

Coronaviruses (CoVs) are a large virus family comprising four genera—*Alphacoronavirus* (α-CoV)*, Betacoronavirus* (β-CoV)*, Gammacoronavirus* (γ-CoV) and *Deltacoronavirus* (δ-CoV). These genera are grouped based on phylogenetic relationship and genomic structure. The β-CoV genera is further grouped into lineages A–D [1].

CoVs infect a wide variety of hosts, some of which include avian species, bats, swine, camels, dogs, cats, bovine, and humans [2,3]. Human CoVs are members of the α-CoV and β-CoV genera with two α-CoVs (NL63 and 229E) and two β-CoVs (HKU1 and OC43) circulating in humans and inducing mild respiratory illnesses such as the common cold [4,5]. Specific spillover events resulting in new CoV infections in humans have occurred in recent history. In 2003, a novel pathogenic coronavirus named severe acute respiratory syndrome coronavirus (SARS-CoV) was linked to transmission events from Himalayan palm civets (*Paguma larvata)* and possibly bats into humans and caused 8096 cases and 774 deaths [6,7,8]. In 2012, another novel coronavirus, Middle East respiratory syndrome coronavirus (MERS-CoV), was linked to spillover from dromedary camels into humans and so far has caused 2553 cases and 876 deaths [9,10,11]. MERS-CoV is closely related to bat CoVs such as PREDICT/PDF-2180 [12,13], suggesting that bats could be an ancestral reservoir. However, antibodies against MERS-CoV have been detected in camel sera obtained 20–30 years ago, arguing that a potential jump from bats to camels occurred several decades ago [14]. SARS-CoV is linked to one major spillover event, while MERS-CoV is still widely circulating among dromedary camels and will continue to transmit to humans without an intervention.

In 2019, a novel CoV emerged in Wuhan, China and resulted in a pandemic. It is hypothesized that SARS-CoV-2 is the result of another spillover event [15]. Malayan pangolins (*Manis javanica*) have been investigated as an intermediate host, but the most likely natural animal reservoir are bats from the *Rhinolophus* genus [16,17,18,19,20,21]. This is the third CoV spillover into the human population in the twenty-first century—highlighting the importance of tracking CoVs in their natural and intermediate reservoirs.

Increased human-to-wildlife interface has raised the risk of CoV spillover events into the human population, and although consensus PCR assays have been widely implemented in programs like PREDICT, they have not been systematically evaluated for sensitivity or updated to account for all the new diversity that has been discovered in recent years. It is important that we better understand CoV dynamics in animal reservoir species as well as reservoir host distribution, density, and prevalence [22]. A well-designed PCR assay allows for detection of every genera of CoVs and identification of the appropriate genera when sequenced. In 2010, Watanabe et al. [23] designed a set of primers that targeted a highly conserved region of the RNA-dependent RNA polymerase (RdRp) gene across all coronavirus genera. Since then, numerous new CoV sequences have been detected and described. Here we provide a modification and validation on the original assay to allow reliable and robust detection of all genera of CoVs. The assay demonstrated high sensitivity and detected CoVs in all genera—including lineage B and C of β-CoVs which include MERS-CoV, SARS-CoV, and SARS-CoV-2. The described novel PCR is suitable for detecting and monitoring CoV circulation in reservoir species.

## 2. Materials and Methods

### 2.1. Primer Design and Optimization

We obtained the sequence of the previously published Watanabe et al. [23] primers 5′-GGTTGGGACTATCCTAAGTGTGA-3′ (Watanabe conventional_F) and 5′-CCATCATCAGATAGAATCATCATA-3′ (Watanabe conventional_R). These primers were mapped to 48 CoVs, which represented all four genera, allowing identification of mismatches. We then redesigned these primers. The primer sequences of the first PCR are: 5′-GGTTGGGAYTAYCCHAARTGYGA-3′ (Pan_CoV_F-1), 5′-CCRTCATCAGAHARWATCAT-3′ (Pan_CoV_R-1), and 5′-CCRTCATCACTHARWATCAT-3′ (Pan_CoV_R-2). The semi-nested second PCR utilizes the same reverse primers and 5′-GAYTAYCCHAARTGTGAYAGA-3′ (Pan_CoV_F-2) and 5′-GAYTAYCCHAARTGTGAYMGH-3′ (Pan_CoV_F-3) as forward primers (Table S1).

### 2.2. Viruses Selected for PCR Establishment

Viruses were either obtained from BEI Resources (https://www.beiresources.org/) or from collaborators. The following CoVs were included: Human CoV NL63 (BEI: NR-470), Canine CoV UCD1 (BEI: NR-868), Porcine Respiratory CoV ISU1 (BEI: NR-454) and Alphacoronavirus purdue P115 (BEI: NR-43285), MERS-CoV (Ron Fouchier, Erasmus EMC), HKU5 pseudo (BEI: NR-48814), 2019-nCoV/SARS-CoV-2 (Natalie Thornburg, CDC), WIV1 (Ralph Baric, UNC), SHC014 (Ralph Baric), Murine CoV icA59 (BEI: NR-43000), HCoV-OC43 (BEI: NR-52725), Bovine CoV Mebus (BEI: NR-445), Bat SARS-like CoV pseudo (BEI: NR-44009), and Avian infectious bronchitis CoV Massachusetts (BEI: NR-43284).

A nucleotide fragment was designed for common moorhen CoV HKU21 based on GenBank reference sequence: NC 016-996. The fragment was transcribed into RNA using the MEGAscript T7 transcription kit (ThermoFischer, Rockville, MD, USA).

### 2.3. RNA Extractions and cDNA Synthesis for Viral Templates

Viral RNA was extracted with the RNeasy Mini Kit (QIAGEN, Hilden, Germany) following the manufacturer’s protocol. cDNA was synthesized using the SuperScript IV First-Strand Synthesis System (ThermoFischer, Rockville, MD, USA). Briefly, 2 μL of 50 ng/µL random hexamers, 1 µL dNTPs (10 mM each), 0.5 µl Ribonuclease Inhibitor (40 U/µL) and 10.5 μL RNA were mixed and incubated for 5 min at 65 °C. 0.5 µL of Ribonuclease Inhibitor (40 U/µL), 4 µL 5× SSIV Buffer, 1 µL 100 mM DTT and 0.5 µL SuperScript^TM^ IV Reverse Transcriptase (200 U/µL) were mixed and 6 µL was added to the reaction. Reverse transcription was performed by incubation for 10 min at 25 °C, 15 min at 50 °C, and 10 min at 80 °C. Finally, 1 µL *E. coli* RNase H was added per reaction and the samples were incubated at 37 °C for 20 min.

### 2.4. Pan-CoV PCR

Optimization of the PCR protocol comprised of evaluating primer sets at different temperatures (49.2 °C–59 °C). It was observed that 48 °C was the most successful in the first PCR and 58 °C was the most successful in the second PCR. The primer concentrations (0.4 µM) remained the same throughout the experimentation process.

An amount of 2 µL of cDNA, 12.5 µL TopTaq Master Mix 2× (QIAGEN), 2.5 µL CoraLoad (QIAGEN), 1 µL of each primer (3 primers, final concentration = 0.4 μM) and 5 µL H_2_O. Thermal cycling for the first round of PCR was performed at 94 °C for 3 min for initial denaturation, followed by 25 cycles of 94 °C for 30 s, 48 °C for 30 s, and 72 °C for one minute, and a final extension at 72 °C for 5 min.

For the newly designed PCR assay, a second amplification was performed using semi-nested primers: 1 µL of PCR product, 12.5 µL TopTaq Master Mix 2× (QIAGEN), 2.5 µL CoraLoad (QIAGEN), 1 µL of each primer (4 primers, final concentration = 0.4 μM) and 5 µL H_2_O. Thermal cycling for the second round of PCR was performed at 94 °C for 3 min, followed by 40 cycles of 94 °C for 30 s, 58 °C for 30 s and 72 °C for 1 min. Visualization of PCR product was done on 2% agarose gel. Water controls were used as negative controls in every PCR run (Table S1).

### 2.5. Limit of Detection (LOD)

cDNA from the 15 in-house CoVs was 10× serially diluted, starting at 1:10 and ending at 1:10,000. Each dilution was assessed 10 times for each assay. The LOD was determined to be the lowest dilution that still resulted in a band on the gel for all replicates. Every individual CoV dilution was tested on a NanoDrop 800 (ThermoFischer, Rockville, Maryland) in duplicate. Concentration (ng/µL) was graphed on a linear scale against the dilution. These mapped values resulted in a unique linear equation (y = ax + b) for each CoV, which was used to calculate copies/µL. If the R^2^ value was less than 0.96 all serially diluted cDNA was remade and retested (Table S2).

### 2.6. Phylogenetic Tree Analysis

In total, 48 CoV sequences representing all genera were chosen and downloaded from NCBI for analysis in Geneious Prime 2019.04. The inner set of primers were mapped to the RdRp, and the theoretical PCR product was determined (~430 nt). This product was aligned and then mapped using ATGC PhyML software [24]. The tree was made using Akaike Information Criterion (AIC) with 1000 bootstraps.

### 2.7. Statistics

Statistical significance was determined using the Spearman’s rank correlation coefficient. Differences were deemed significant when *p*-value was <0.05.

## 3. Results

### 3.1. Primer Design

**Figure 1:**
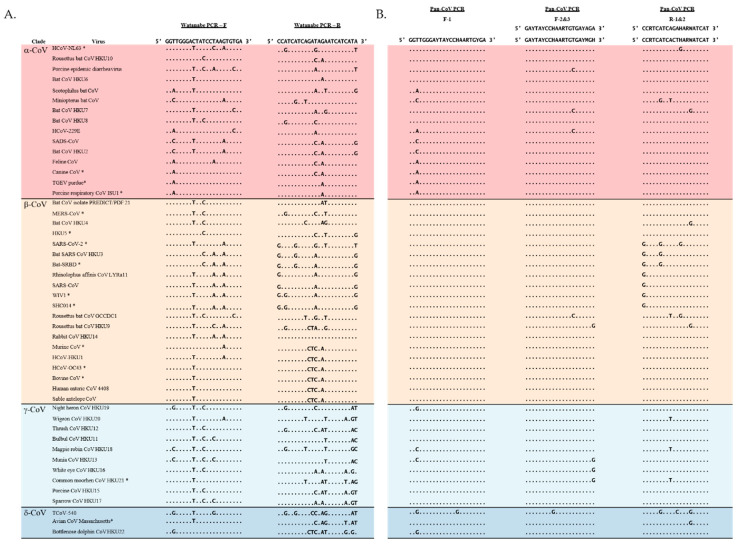
Primer aligned with 48 CoV sequences that represent all four genera of CoVs. Representatives for each group were chosen randomly and include all tested CoVs. (**A**) Watanabe and (**B**) Pan-CoV primers aligned with CoV representatives. Red: α-CoVs, yellow: β−CoVs, light blue: δ-CoVs, dark blue: γ-CoVs. * = in-house tested CoVs.

The Watanabe and newly designed primers were aligned to 48 CoVs which included 16 α-CoVs, 19 β-CoVs, 10 γ-CoVs and 3 δ-CoVs (Figure 1). The Pan-CoV forward primers used the same location as the Watanabe forward primers. The Pan_CoV_R-1 and R-2 were designed ~440 nt away from Pan_CoV_F-1. Semi-nested forward primers were designed to increase sensitivity. Degenerate bases were added to areas with high sequence variability to increase the likelihood of detecting CoVs. Two reverse primers were designed to ensure less than five degenerate bases per primer. The pan-CoV outer primer set results in a ~440 nt product and the semi-nested inner primer set results in a ~430 nt product. The Watanabe and Pan-CoV PCR primers were mapped to a reference genome (MERS-CoV: MF598663.1) and the genome location is denoted by nucleotide numbers (Table S4).

### 3.2. Pan-CoV PCR Assay Limit of Detection

**Figure 2:**
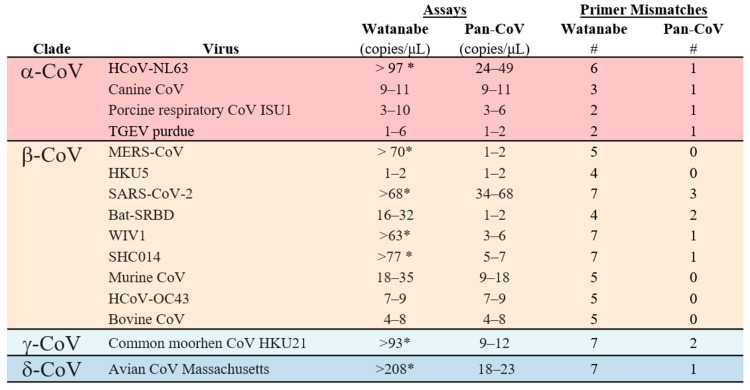
Limit of detection (LOD) for the Watanabe and Pan-CoV PCR assays. The LOD was determined for both assays for 15 CoVs from four genera. # = the total number of primer mismatches. * = not detected in assay.

**Figure 3:**
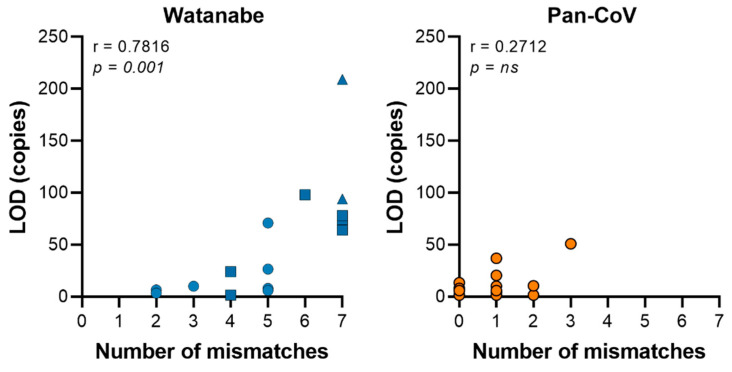
The sum of mismatches in the primer set contribute to the sensitivity of the PCR assay. Graph displaying the sum of mismatches within primer sets against the average limit of detection (LOD) per virus. A significant correlation was found for the Watanabe primers, but not the Pan-CoV primers using a Spearman’s rank correlation coefficient. Circle = no mismatches in 3′ end primer; square = 1 mismatch in 3′ end primer; triangle = >1 mismatch in 3′ end primer.

After computational optimization, both primer sets were compared with regards to sensitivity and breadth of CoV detection. Both the Watanabe primers and Pan-CoV primers were compared against 15 CoVs; 4 α−CoVs, 9 β-CoVs, 1 γ-CoV and 1 δ-CoV. The Pan-CoV primers successfully detected all of the in-house CoVs with a sensitivity range of 1-68 copies/µL. The Watanabe primers did not detect seven of the 15 in-house CoVs. In our hands and at the highest concentration used, the Watanabe primers were unable to detect human CoV NL63, MERS-CoV, SARS-CoV-2, WIV1, SHC014, common moorhen CoV HKU21, and Avian CoV Massachusetts (Table S3). For the CoVs recognized, we found that both primer sets achieved a sensitivity of between 1–100 copies/μL. Overall, the newly designed assay was more sensitive than the original assay (Figure 2). We then analyzed correlation between the sum of mismatches in primers and the LOD (Figure 3). A significant correlation was found for the Watanabe primers (r = 0.7816, *p* = 0.001), but not the pan-CoV primers (r = 0.2712, *p* = 0.3270).

### 3.3. Phylogenetic Tree Analysis

**Figure 4:**
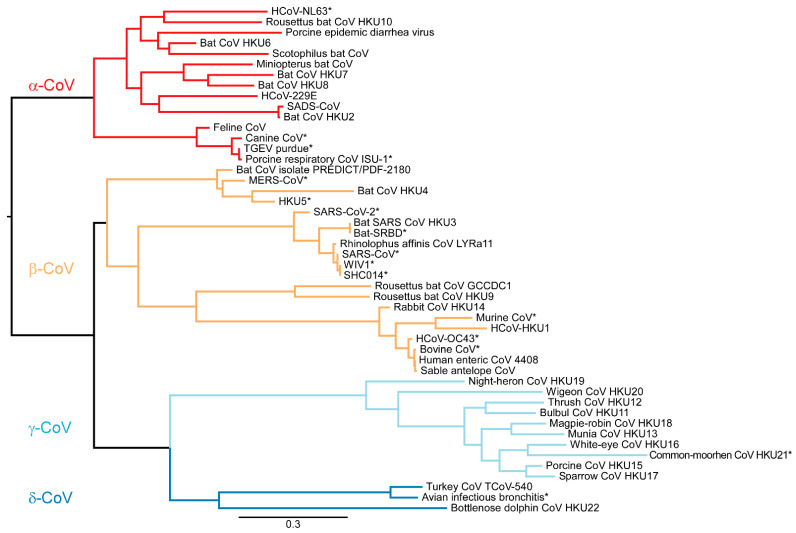
Maximum likelihood phylogenetic tree analysis with 1000 bootstraps made in PhyML [24] displays the 430 nt product from the Pan-CoV PCR. The PCR product allows for separation into four CoV genera. * = tested CoVs in Pan-CoV PCR.

The sequences used for the phylogenetic tree were obtained *in silico* by mapping the primers to 48 CoV sequences and determining the theoretical Pan-CoV PCR product. These sequences were aligned and mapped using ATGC PhyML software. Based on the theoretical 430 nt product, we observed clustering of the four genera of CoVs: α-CoV, β-CoV, γ-CoV and δ-CoV (Figure 4). We thus hypothesize that the sequence of the Pan-CoV PCR product will allow identification of the genera (α-CoV, β-CoV, γ-CoV, and δ-CoV), including lineages A–D of the β-CoV genera.

## 4. Discussion

The availability of a sensitive and easy-to-use assay to detect coronaviruses is important to better understand the prevalence of coronavirus in different animal host. When comparing the Watanabe assay against the newly designed assay which was developed as a nested PCR assay, our assay was able to detect a wider range of CoVs with comparable sensitivity. This is likely because of the optimization of primers. The Watanabe primer sets were less sensitive in detecting coronavirus when there were more than four mismatches in the primers. We found that for the Watanabe assay, an increasing number of mismatches significantly correlated with an increase in the LOD. We did not find such a correlation for the Pan-CoV assay (Figure 1). Importantly, the Watanabe primers contained mismatches towards the 3′ end of the reverse primer of some CoVs, which likely contributed to the observed reduced sensitivity. In contrast, our newly designed assay was more sensitive in detecting all of the coronaviruses tested, including lineage B and C of the β-CoV genera which contain SARS-CoV, SARS-CoV-2, and MERS-CoV. Based on the small PCR product, all CoVs separated into their appropriate genera down to the β-CoV specific lineages. The Pan-CoV PCR product can be sequenced using the Pan-CoV semi-nested primers. As demonstrated by the *in silico* results, this sequence will allow placement in the CoV phylogenetic tree. Based on this result, one can decide to perform full genome sequencing using Illumina and Next Generation Sequencing. With the recent emergence of SARS-CoV-2, increased research efforts will be focused at the origin and zoonotic potential of CoVs. Sensitive pan-CoV assays will be crucial in order to study the coronavirus diversity in natural reservoirs, production animals and wet markets. We would expect to detect γ-CoVs and δ-CoVs predominately in avian species [25]. In monkey, feline, canine, rodent, and bat species, we would expect to see α-CoVs and β-CoVs [26].

Our modified assay is semi-nested, whereas the original Watanabe assay used a single PCR. Although sensitivity of a semi-nested PCR is higher, there are several drawbacks to this approach: the method is more time-consuming, more costly and most importantly the chance of contamination is higher. When we compared the results of the Watanabe assay to the results of our first PCR (Table S3), we noticed an improvement in the ability to detect CoVs (Watanabe detected 8 out of 15, Pan CoV detected 14 out of 15). Thus, if one prefers not to utilize a semi-nested PCR, the redesigned primers will still provide an improvement for single PCR.

In conclusion, our assay was able to reliably detect all in-house CoVs, which represented the four CoV genera. This could be an important tool in detecting potential spillover events from reservoir hosts. The PCR product can be sequenced to identify known and novel CoV strains and track circulation in hosts. The Pan-CoV PCR, demonstrated in this paper, will provide a resource for future research to identify novel CoVs, monitor circulation CoVs and detect potential spillover events.

## Data Availability

Data is contained within the article or supplementary material.

## Supplementary Materials

The following are available online at https://www.mdpi.com/article/10.3390/v13040599/s1, Table S1: Pan-CoV PCR protocol with primers and thermocycling conditions. Table S2: Scatter plots and linear equations for in-house CoV cDNA. Table S3: Gels showing the seven tested CoVs detected by the Pan-CoV primers but not by the Watanabe primers. Table S4: Placing primers in MERS-CoV reference sequence. Table S5: Acknowledgements for the sequences used.
