# Correction: Holbrook et al. Updated and Validated Pan-Coronavirus PCR Assay to Detect All Coronavirus Genera. *Viruses* 2021, *13*, 599

**DOI:** 10.3390/v17101364

**Published:** 2025-10-13

**Authors:** Myndi G. Holbrook, Simon J. Anthony, Isamara Navarrete-Macias, Theo Bestebroer, Vincent J. Munster, Neeltje van Doremalen

**Affiliations:** 1 Laboratory of Virology, National Institute of Allergy and Infectious Diseases, National Institutes of Health, Hamilton, MT 59840, USA; myndi.holbrook@nih.gov (M.G.H.); vincent.munster@nih.gov (V.J.M.); 2 Department of Pathology, Microbiology, & Immunology, School of Veterinary Medicine, University of California, Davis, Davis, CA 95616, USA; sjanthony@ucdavis.edu (S.J.A.); i.navarrete.macias@gmail.com (I.N.-M.); 3 Department of Viroscience, Erasmus MC Rotterdam, 3015 GE Rotterdam, The Netherlands; t.bestebroer@erasmusmc.nl

There was an error in the original publication [1]. In the Materials and Methods Section, the sequence of the Pan-CoV_F-1 primer is missing a T, but it is shown correctly in the Supplementary Material.

A correction has been made to 2. Materials and Methods, 2.1 Primer Design and Optimization:

2.1. Primer Design and Optimization

We obtained the sequence of the previously published Watanabe et al. [23] primers 5′-GGTTGGGACTATCCTAAGTGTGA-3′ (Watanabe conventional_F) and 5′-CCATCATCAGATAGAATCATCATA-3′ (Watanabe conventional_R). These primers were mapped to 48 CoVs, which represented all four genera, allowing identification of mismatches. We then redesigned these primers. The primer sequences of the first PCR are: 5′-GGTTGGGAYTAYCCHAARTGYGA-3′ (Pan_CoV_F-1), 5′-CCRTCATCAGAHARWATCAT-3′ (Pan_CoV_R-1), and 5′-CCRTCATCACTHARWATCAT-3′ (Pan_CoV_R-2). The semi-nested second PCR utilizes the same reverse primers and 5′-GAYTAYCCHAARTGTGAYAGA-3′ (Pan_CoV_F-2) and 5′-GAYTAYCCHAARTGTGAYMGH-3′ (Pan_CoV_F-3) as forward primers (Table S1).

The authors state that the scientific conclusions are unaffected. This correction was approved by the Academic Editor. The original publication has also been updated.
