# Supplementary material for: Updated and Validated Pan-Coronavirus PCR Assay to Detect All Coronavirus Genera
Source: Viruses. 2021 Apr 1;13(4):599. doi: 10.3390/v13040599 (PMC8067199; doi:10.3390/v13040599)
Supplement: Supplementary file 1 [file viruses-13-00599-s001.pdf]

Table S1. Pan-CoV PCR protocol with primers and thermocycling conditions.

Pan-CoV PCR 1: Pan_CoV_F1 + Pan_CoV_R1 + Pan_CoV_R2
Pan-CoV PCR 2: Pan_CoV_R1 + Pan_CoV_R2 + Pan-CoV_F2 + Pan_CoV_F3

Primers diluted: 190 µl water + 10 µl primer (200 µM)

Pan_CoV_F1	GGTTGGGAYTAYCCHAARTGYGA
Pan_CoV_R1	CCRTCATCAGAHARWATCAT
Pan_CoV_R2	CCRTCATCACTHARWATCAT
Pan_CoV_F2	GAYTAYCCHAARTGTGAYAGA
Pan_CoV_F3	GAYTAYCCHAARTGTGAYMGH

1st PCR: Pan-CoV PCR 1

**Total**	**25 µl**	**Thermocycling conditions:**
Pan_CoV_F1 (10 pmol/ µl)	1 µl	Incubate 3 min 94°C<br>25 cycles (30 sec 94°C, 30 sec 48°C, 1 min 72°C)<br>5 min 72°C
Pan_CoV_R1 (10 pmol/ µl)	1 µl	
Pan_CoV_R2 (10 pmol/ µl)	1 µl	
TopTaq Master Mix	12.5 µl	
Water	5 µl	
CoraLoad	2.5 µl	
cDNA	2 µl	

2nd PCR: Pan-CoV PCR 2

**Total**	**25 µl**	**Thermocycling conditions:**
Pan_CoV_R1 (10 pmol/ µl)	1 µl	Incubate 3 min 94°C<br>40 cycles (30 sec 94°C, 30 sec 58°C, 1 min 72°C)<br>5 min 72°C
Pan_CoV_R2 (10 pmol/ µl)	1 µl	
Pan_CoV_F2 (10 pmol/ µl)	1 µl	
Pan_CoV_F3 (10 pmol/ µl)	1 µl	
TopTaq Master Mix	12.5 µl	
Water	5 µl	
CoraLoad	2.5 µl	
Product first PCR	1 µl	

*Run results on 2% gel

Table S2. Scatter plots and linear equations for in-house CoV cDNA (A) Raw reads from spectrometer on serially diluted cDNA for all in-house CoVs (B) Scatter plots graphed for each CoV with linear equation

A.

Sample ID	Conc.	Units	A260	A280	260/280	260/230	Conc. Factor (ng/ul)	Cursor Pos.	Cursor abs.	340 raw	NA Type
HCoV-NL63 1:10	97.49	ng/ul	2.954	1.66	1.78	1.64	33	260	2.954	0.073	ssDNA-33
	100	ng/ul	3.03	1.691	1.79	1.65	33	260	3.03	0.068	ssDNA-33
HCoV-NL63 1:100	9.878	ng/ul	0.299	0.162	1.85	1.8	33	260	0.299	0.056	ssDNA-33
	9.687	ng/ul	0.294	0.143	2.06	1.87	33	260	0.294	0.048	ssDNA-33
HCoV-NL63 1:1,000	0.8648	ng/ul	0.026	0.014	1.81	1.15	33	260	0.026	0.052	ssDNA-33
	1.189	ng/ul	0.036	0.034	1.06	1.11	33	260	0.036	0.046	ssDNA-33
HCoV-NL63 1:10,000	0.1466	ng/ul	0.004	-0.002	-2.31	-1.08	33	260	0.004	0.062	ssDNA-33
	-0.3785	ng/ul	-0.011	0	132	1.25	33	260	-0.011	0.059	ssDNA-33
Canine CoV 1:10	91.85	ng/ul	2.783	1.58	1.76	1.61	33	260	2.783	0.028	ssDNA-33
	91.12	ng/ul	2.761	1.567	1.76	1.59	33	260	2.761	0.016	ssDNA-33
Canine CoV 1:100	10.49	ng/ul	0.318	0.171	1.86	1.63	33	260	0.318	0.006	ssDNA-33
	10.13	ng/ul	0.307	0.172	1.79	1.77	33	260	0.307	0.024	ssDNA-33
Canine CoV 1:1,000	0.5521	ng/ul	0.017	-0.002	-9.56	1.34	33	260	0.017	0.016	ssDNA-33
	0.7519	ng/ul	0.023	0.002	10.85	-3.43	33	260	0.023	0.013	ssDNA-33
Canine CoV 1:10,000	-0.8313	ng/ul	-0.025	-0.028	0.91	1.51	33	260	-0.025	0.023	ssDNA-33
	-0.2181	ng/ul	-0.007	-0.016	0.42	1.26	33	260	-0.007	0.008	ssDNA-33
Porcine respiratory CoV ISU 1:10	85.46	ng/ul	2.59	1.454	1.78	1.41	33	260	2.591	0.016	ssDNA-33
	85.72	ng/ul	2.598	1.436	1.81	1.42	33	260	2.598	0.008	ssDNA-33
Porcine respiratory CoV ISU 1:100	12.43	ng/ul	0.377	0.225	1.68	1.52	33	260	0.377	-0.006	ssDNA-33
	12.06	ng/ul	0.365	0.228	1.6	1.54	33	260	0.366	0.012	ssDNA-33
Porcine respiratory CoV ISU 1:1,000	0.9296	ng/ul	0.028	0.026	1.1	1.39	33	260	0.029	-0.003	ssDNA-33
	1.479	ng/ul	0.045	0.029	1.56	3.04	33	260	0.045	0	ssDNA-33
Porcine respiratory CoV ISU 1:10,000	0.3263	ng/ul	0.01	0.021	0.48	-1.44	33	260	0.011	0.001	ssDNA-33
	0.1193	ng/ul	0.004	0.01	0.37	-0.33	33	260	0.004	-0.006	ssDNA-33
TGEV Purdue P115 1:10	59.46	ng/ul	1.802	1.026	1.76	1.55	33	260	1.802	0.024	ssDNA-33
	59.66	ng/ul	1.808	1.029	1.76	1.53	33	260	1.808	0.021	ssDNA-33
TGEV Purdue P115 1:100	6.681	ng/ul	0.202	0.113	1.79	1.7	33	260	0.202	0.022	ssDNA-33
	6.166	ng/ul	0.187	0.1	1.87	1.56	33	260	0.187	0.016	ssDNA-33
TGEV Purdue P115 1:1,000	0.7659	ng/ul	0.023	0.003	8.09	1.38	33	260	0.023	0.003	ssDNA-33

TGEV Purdue P115 1:10,000	0.3049	ng/ul	0.009	-0.001	-6.23	0.79	33	260	0.009	0.007	ssDNA-33
	0.2087	ng/ul	0.006	0.008	0.8	0.35	33	260	0.006	0.025	ssDNA-33
	0.2417	ng/ul	0.007	0.011	0.67	0.26	33	260	0.007	0.015	ssDNA-33
MERS-CoV 1:10	68.95	ng/ul	2.089	1.175	1.78	1.17	33	260	2.089	0.089	ssDNA-33
	66.59	ng/ul	2.018	1.116	1.81	1.2	33	260	2.018	0.081	ssDNA-33
MERS-CoV 1:100	7.237	ng/ul	0.219	0.143	1.53	1.17	33	260	0.219	-0.015	ssDNA-33
	7.155	ng/ul	0.217	0.138	1.57	1.35	33	260	0.217	0.002	ssDNA-33
MERS-CoV 1:1,000	0.762	ng/ul	0.023	0.019	1.22	0.74	33	260	0.023	-0.007	ssDNA-33
	0.9321	ng/ul	0.028	0.043	0.66	0.86	33	260	0.028	-0.01	ssDNA-33
MERS-CoV 1:10,000	0.3111	ng/ul	0.009	0.027	0.35	0.62	33	260	0.009	-0.013	ssDNA-33
	0.269	ng/ul	0.008	0	-82.92	1.07	33	260	0.008	-0.012	ssDNA-33
HKU5 1:10	75.72	ng/ul	2.295	1.288	1.78	1.68	33	260	2.293	0.011	ssDNA-33
	82.1	ng/ul	2.488	1.404	1.77	1.7	33	260	2.49	0.012	ssDNA-33
HKU5 1:100	8.871	ng/ul	0.269	0.169	1.59	1.72	33	260	0.27	-0.002	ssDNA-33
	8.473	ng/ul	0.257	0.16	1.6	1.87	33	260	0.258	-0.006	ssDNA-33
HKU5 1:1,000	0.9817	ng/ul	0.03	0.037	0.8	4.71	33	260	0.031	-0.005	ssDNA-33
	1.173	ng/ul	0.036	0.04	0.89	1.12	33	260	0.037	-0.01	ssDNA-33
HKU5 1:10,000	0.1067	ng/ul	0.003	0.024	0.14	6.92	33	260	0.005	0.005	ssDNA-33
	0.1421	ng/ul	0.004	0.019	0.23	0.38	33	260	0.005	-0.014	ssDNA-33
SARS-CoV-2 1:10	66.43	ng/ul	2.013	1.098	1.83	1.85	33	260	2.013	0.07	ssDNA-33
	68.93	ng/ul	2.089	1.135	1.84	1.87	33	260	2.089	0.069	ssDNA-33
SARS-CoV-2 1:100	6.726	ng/ul	0.204	0.103	1.99	2.84	33	260	0.204	0.029	ssDNA-33
	6.457	ng/ul	0.196	0.101	1.93	2.32	33	260	0.196	0.033	ssDNA-33
SARS-CoV-2 1:1,000	0.717	ng/ul	0.022	0.011	1.93	-0.81	33	260	0.022	0.032	ssDNA-33
	0.2262	ng/ul	0.007	-0.009	-0.72	-0.27	33	260	0.007	0.046	ssDNA-33
SARS-CoV-2 1:10,000	-0.5315	ng/ul	-0.016	-0.02	0.82	0.36	33	260	-0.016	0.042	ssDNA-33
	-0.6915	ng/ul	-0.021	-0.017	1.22	0.4	33	260	-0.021	0.046	ssDNA-33
WIV1 1:10	63.32	ng/ul	1.919	1.122	1.71	1.83	33	260	1.919	0.021	ssDNA-33
	62.8	ng/ul	1.903	1.108	1.72	1.79	33	260	1.903	0.031	ssDNA-33
WIV1 1:100	6.103	ng/ul	0.185	0.12	1.54	1.86	33	260	0.185	0.007	ssDNA-33
	5.91	ng/ul	0.179	0.109	1.64	1.71	33	260	0.179	0.001	ssDNA-33
WIV1 1:1,000	0.4116	ng/ul	0.012	0.015	0.82	1.26	33	260	0.012	0.001	ssDNA-33
	0.5877	ng/ul	0.018	0.014	1.31	0.75	33	260	0.018	-0.002	ssDNA-33

WIV1 1:10,000	0.7635	ng/ul	0.023	0.017	1.39	0.87	33	260	0.023	-0.005	ssDNA-33
	0.856	ng/ul	0.026	0.025	1.05	0.52	33	260	0.026	0.005	ssDNA-33
SHC014 1:10	76.75	ng/ul	2.326	1.346	1.73	1.88	33	260	2.326	0.015	ssDNA-33
	77.31	ng/ul	2.343	1.364	1.72	1.88	33	260	2.343	0.008	ssDNA-33
SHC014 1:1,000	0.7298	ng/ul	0.022	0.013	1.73	1.56	33	260	0.022	-0.001	ssDNA-33
	0.345	ng/ul	0.01	0.007	1.51	-1.22	33	260	0.01	0.007	ssDNA-33
SHC014 1:10,000	-0.4287	ng/ul	-0.013	-0.008	1.6	4.68	33	260	-0.013	0.008	ssDNA-33
	0.3286	ng/ul	0.01	0.016	0.63	3.37	33	260	0.01	-0.012	ssDNA-33
Murine CoV 1:10	78.43	ng/ul	2.377	1.367	1.74	1.29	33	260	2.377	0.033	ssDNA-33
	79.67	ng/ul	2.414	1.365	1.77	1.32	33	260	2.414	0.032	ssDNA-33
Murine CoV 1:100	9.44	ng/ul	0.286	0.162	1.77	1.83	33	260	0.286	0.041	ssDNA-33
	9.026	ng/ul	0.274	0.152	1.8	1.62	33	260	0.274	0.045	ssDNA-33
Murine CoV 1:1,000	0.6179	ng/ul	0.019	0.009	1.99	-0.52	33	260	0.019	0.041	ssDNA-33
	0.4813	ng/ul	0.015	0.002	7.02	-0.98	33	260	0.015	0.042	ssDNA-33
Murine CoV 1:10,000	-0.1369	ng/ul	-0.004	-0.01	0.4	0.11	33	260	-0.004	0.038	ssDNA-33
	-0.248	ng/ul	-0.008	-0.005	1.58	0.21	33	260	-0.008	0.04	ssDNA-33
HCoV-OC43 1:10	70.75	ng/ul	2.144	1.211	1.77	1.73	33	260	2.144	0.006	ssDNA-33
	70.55	ng/ul	2.138	1.213	1.76	1.69	33	260	2.138	-0.011	ssDNA-33
HCoV-OC43 1:100	7.267	ng/ul	0.22	0.134	1.64	1.98	33	260	0.22	0.006	ssDNA-33
	6.919	ng/ul	0.21	0.129	1.62	1.69	33	260	0.21	0.008	ssDNA-33
HCoV-OC43 1:1,000	0.656	ng/ul	0.02	0.01	2.07	1.37	33	260	0.02	-0.007	ssDNA-33
	0.7791	ng/ul	0.024	0.03	0.79	0.73	33	260	0.024	0.008	ssDNA-33
HCoV-OC43 1:10,000	-0.1723	ng/ul	-0.005	0.013	-0.4	-2.95	33	260	-0.005	0.006	ssDNA-33
	-0.2433	ng/ul	-0.007	-0.001	6.61	1.79	33	260	-0.007	-0.001	ssDNA-33
Bovine CoV 1:10	80.64	ng/ul	2.444	1.387	1.76	1.65	33	260	2.442	0.069	ssDNA-33
	83.67	ng/ul	2.535	1.432	1.77	1.69	33	260	2.534	0.006	ssDNA-33
Bovine CoV 1:1,000	7.944	ng/ul	0.241	0.138	1.74	1.83	33	260	0.242	-0.002	ssDNA-33
	7.436	ng/ul	0.225	0.143	1.58	1.89	33	260	0.225	0.006	ssDNA-33
Bovine CoV 1:10,000	0.05363	ng/ul	0.002	0.015	0.11	0.24	33	260	0.002	-0.011	ssDNA-33
	-0.3351	ng/ul	-0.01	0.011	-0.97	1.47	33	260	-0.01	-0.004	ssDNA-33
Bat SRBD 1:10	95.46	ng/ul	2.893	1.653	1.75	1.55	33	260	2.892	0.032	ssDNA-33
	98.25	ng/ul	2.977	1.692	1.76	1.54	33	260	2.977	0.056	ssDNA-33
Bat SRBD 1:100	10.26	ng/ul	0.311	0.185	1.68	1.78	33	260	0.31	-0.003	ssDNA-33

	10.57	ng/ul	0.32	0.197	1.63	1.69	33	260	0.322	-0.004	ssDNA-33
Bat SRBD 1:1,000	0.9923	ng/ul	0.03	0.029	1.03	3.75	33	260	0.029	-0.012	ssDNA-33
	1.133	ng/ul	0.034	0.031	1.1	2.16	33	260	0.035	0.002	ssDNA-33
Bat SRBD 1:10,000	0.2041	ng/ul	0.006	0.016	0.38	-0.77	33	260	0.005	0.007	ssDNA-33
	0.1272	ng/ul	0.004	0.008	0.48	-0.3	33	260	0.002	-0.008	ssDNA-33
Common Moorhen CoV HKU21 1:10	93.33	ng/ul	2.828	1.59	1.78	1.91	33	260	2.828	0.022	ssDNA-33
	93.22	ng/ul	2.825	1.588	1.78	1.94	33	260	2.825	0.015	ssDNA-33
Common Moorhen CoV HKU21 1:100	10.51	ng/ul	0.319	0.186	1.71	1.91	33	260	0.319	-0.009	ssDNA-33
	9.874	ng/ul	0.299	0.181	1.65	2.07	33	260	0.299	0.002	ssDNA-33
Common Moorhen CoV HKU21 1:1,000	0.7384	ng/ul	0.022	0.011	1.99	-5.18	33	260	0.022	-0.011	ssDNA-33
	0.3899	ng/ul	0.012	0.004	3.2	2.87	33	260	0.012	0.003	ssDNA-33
Common Moorhen CoV HKU21 1:10,000	-0.2889	ng/ul	-0.009	0.002	-4.82	0.63	33	260	-0.009	0.001	ssDNA-33
	-0.3985	ng/ul	-0.012	0.001	-10.17	2.56	33	260	-0.012	-0.012	ssDNA-33
Avian CoV Massachusetts 1:10	211	ng/ul	6.393	3.574	1.79	1.66	33	260	6.393	0.093	ssDNA-33
	204.8	ng/ul	6.208	3.457	1.8	1.68	33	260	6.208	0.072	ssDNA-33
Avian CoV Massachusetts 1:100	14.25	ng/ul	0.432	0.243	1.78	1.92	33	260	0.432	0.036	ssDNA-33
	14.35	ng/ul	0.435	0.242	1.8	2.06	33	260	0.435	0.046	ssDNA-33
Avian CoV Massachusetts 1:1,000	1.071	ng/ul	0.032	0.01	3.39	-0.85	33	260	0.032	0.057	ssDNA-33
	0.8279	ng/ul	0.025	0.013	1.88	-0.8	33	260	0.025	0.048	ssDNA-33
Avian CoV Massachusetts 1:10,000	0.2264	ng/ul	0.007	0	-7046.97	-0.28	33	260	0.007	0.05	ssDNA-33
	-0.2182	ng/ul	-0.007	-0.007	0.94	0.18	33	260	-0.007	0.03	ssDNA-33

B.

**Virus**	
HCoV-NL63	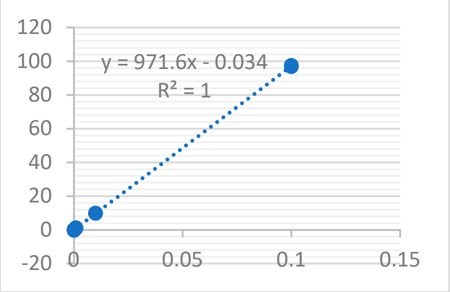

Canine CoV	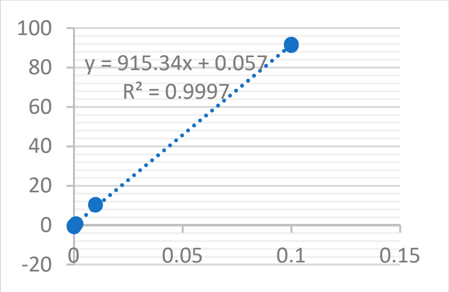
Porcine respiratory CoV ISU1	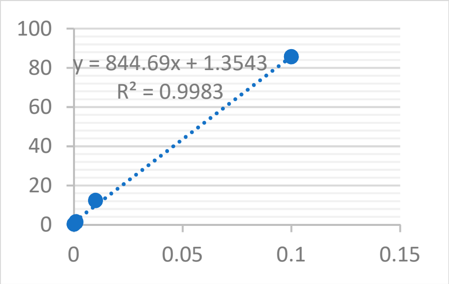
TGEV Purdue	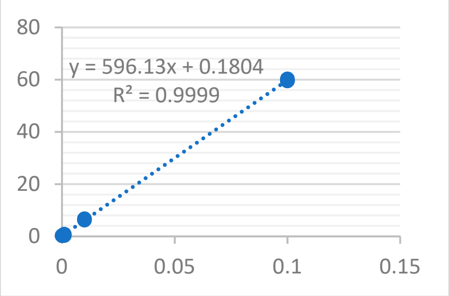
MERS-CoV	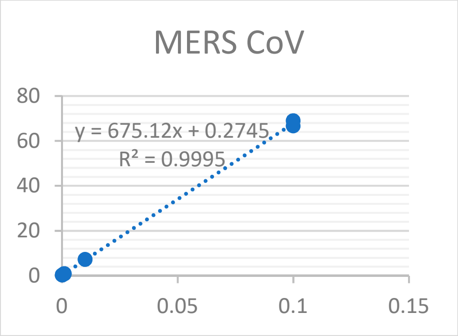
HKU5	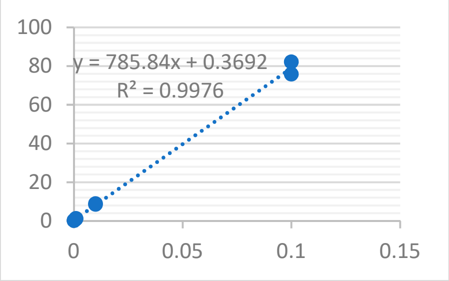
SARS-CoV-2	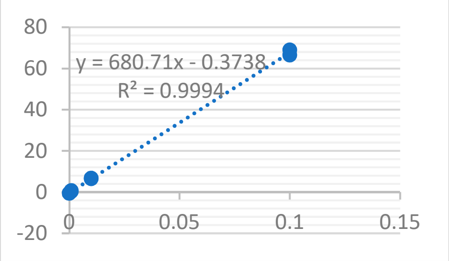

WIV1	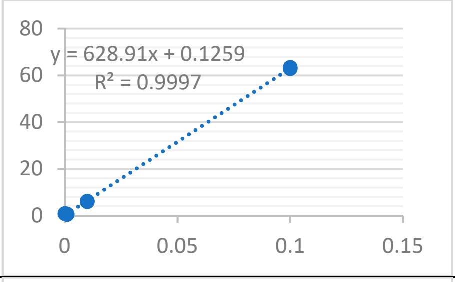
SHC014	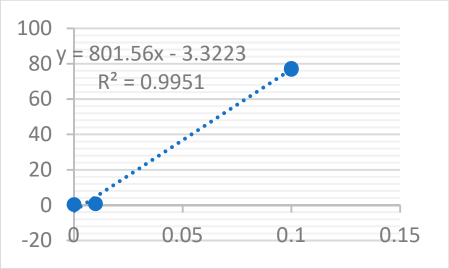
Murine CoV	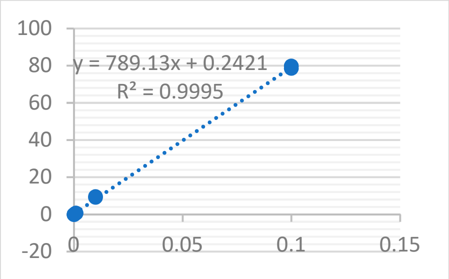
HCoV-OC43	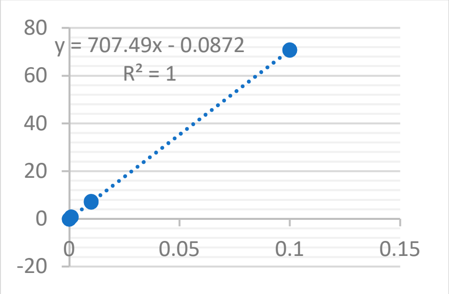
Bovine CoV	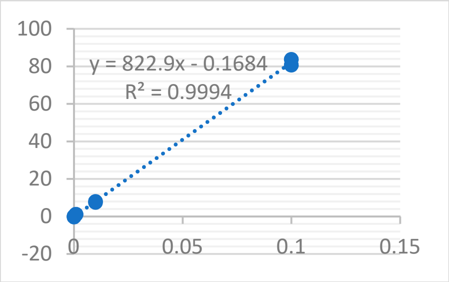
Bat SRBD	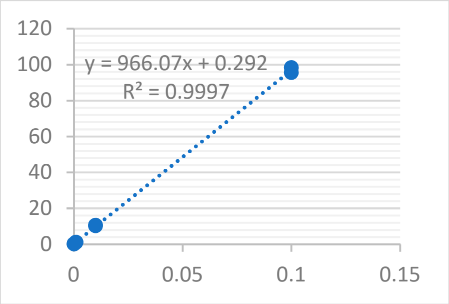

Common Moorhen CoV HKU21	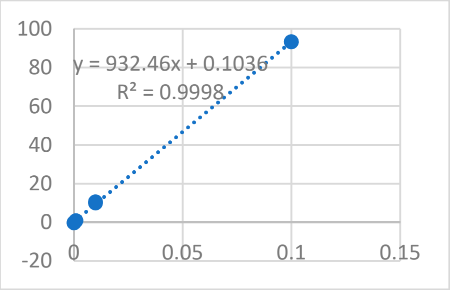
Avian CoV Massachusetts	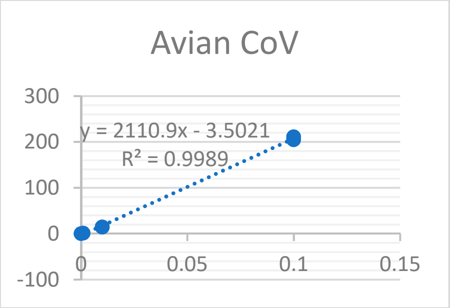

Table S3: Gels showing the seven tested CoVs detected by the Pan-CoV primers but not by the Watanabe primers. These are demonstrating a 10-fold dilution in duplicate. * = expected PCR size

Clade	Virus	Watanabe PCR	Pan-CoV PCR	Pan-CoV PCR (1st round PCR)
α-CoV	HCoV-NL63	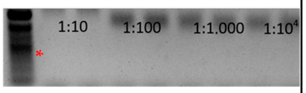	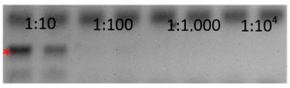	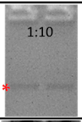
β-CoV	MERS-CoV	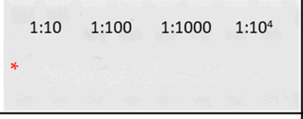	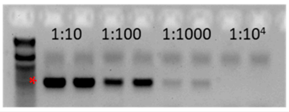	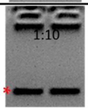
	SARS-CoV-2	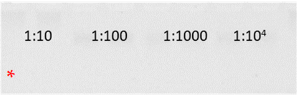	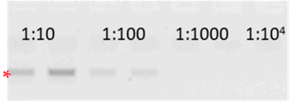	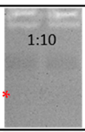
	WIV1	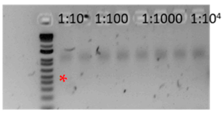	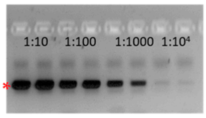	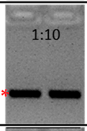
	SHC014	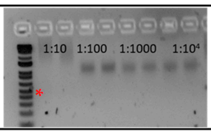	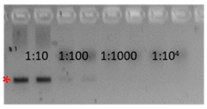	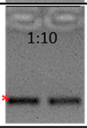
γ-CoV	Common moorhen CoV HKU21	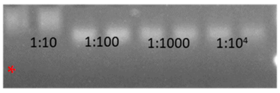	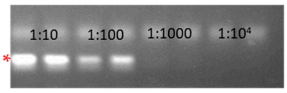	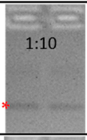
δ-CoV	Avian CoV Massachusetts	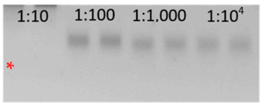	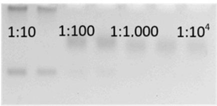	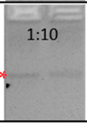

Table S4. Placing primers in MERS-CoV reference sequence. A reference MERS-CoV genome was used to map the Watanabe and Pan-CoV primers, genome location is denoted by nucleotide numbers.

MERS-CoV genome: MF598663.1	
**Primer name**	**Nucleotide #**
Watanabe PCR - F	15257-15,279
Watanabe PCR - R	15,673-15,696
Pan-CoV F-1	15,257-15,279
Pan-CoV F-2&3	15,263-15,283
Pan-CoV R-1&2	16,677-15,696

Table S5: Acknowledgements for the sequences used.

Virus name	Accession No.	Collected	Authors
Porcine epidemic diarrhea virus	NC_003436.1	13-Aug-18	Kocherhans,R., Bridgen,A., Ackermann,M. and Tobler,K.
Scotophilus bat CoV	NC_009657.1	13-Aug-18	Tang,X.C., Zhang,J.X., Zhang,S.Y., Wang,P., Fan,X.H., Li,L.F.,Li,G., Dong,B.Q., Liu,W., Cheung,C.L., Xu,K.M., Song,W.J.,Vijaykrishna,D., Poon,L.L., Peiris,J.S., Smith,G.J., Chen,H. and Guan,Y.
Bat CoV HKU6	DQ249224.1	26-Jul-16	Woo,P.C., Lau,S.K., Li,K.S., Poon,R.W., Wong,B.H., Tsoi,H.W.,Yip,B.C., Huang,Y., Chan,K.H. and Yuen,K.Y.
Miniopterus bat CoV	EU420138.1	21-Feb-18	Chu,D.K., Peiris,J.S., Chen,H., Guan,Y. and Poon,L.L.
Bat CoV HKU8	NC_010438.1	13-Aug-18	Chu,D.K., Peiris,J.S., Chen,H., Guan,Y. and Poon,L.L.
Bat CoV HKU7	DQ249226.1	26-Jul-16	Woo,P.C., Lau,S.K., Li,K.S., Poon,R.W., Wong,B.H., Tsoi,H.W.,Yip,B.C., Huang,Y., Chan,K.H. and Yuen,K.Y.
HCoV-229E	KU291448.1	4-Sep-16	Corman,V.M., Eckerle,I., Memish,Z.A., Liljander,A.M., Dijkman,R., Jonsdottir,H., Juma Ngeiywa,K.J., Kamau,E., Younan,M., Al Masri,M., Assiri,A., Gluecks,I., Musa,B.E., Meyer,B., Muller,M.A., Hilali,M., Bornstein,S., Wernery,U., Thiel,V., Jores,J., Drexler,J.F. and Drosten,C.
HCoV-NL63	AY567487.2	22-Jun-04	van der Hoek,L., Pyrc,K., Jebbink,M.F., Vermeulen-Oost,W.,Berkhout,R.J.M., Wolthers,K.C., Wertheim-van Dillen,P.M.E., Kaandorp,J., Spaargaren,J. and Berkhout,B.
Rousettus bat CoV HKU10	NC_018871.1	13-Aug-18	Lau,S.K., Li,K.S., Tsang,A.K., Shek,C.T., Wang,M., Choi,G.K.,Guo,R., Wong,B.H., Poon,R.W., Lam,C.S., Wang,S.Y., Fan,R.Y., Chan,K.H., Zheng,B.J., Woo,P.C. and Yuen,K.Y.
Bat CoV HKU2	NC_009988.1	13-Aug-18	Lau,S.K., Li,K.S., Tsang,A.K., Shek,C.T., Wang,M., Choi,G.K.,Guo,R., Wong,B.H., Poon,R.W., Lam,C.S., Wang,S.Y., Fan,R.Y., Chan,K.H., Zheng,B.J., Woo,P.C. and Yuen,K.Y.
SADS-CoV	MG605091.1	16-Sep-18	Zhou,L., Sun,Y., Lan,T., Wu,R.T., Chen,J.W., Wu,Z.X., Xie,Q.M.,Zhang,X.B. and Ma,J.Y.
Feline CoV	KX722529.1	31-Dec-17	Ryt-Hansen,P., Krog,J.S. and Larsen,L.E.
Canine CoV	KP981644.1	28-May-15	Decaro,N., Mari,V., Dowgier,G., Elia,G., Lanave,G., Colaianni,M.L. and Buonavoglia,C.
Porcine respiratory CoV ISU1	DQ811787.1	14-Jul-16	Zhang,X., Hasoksuz,M., Spiro,D., Halpin,R., Wang,S., Stollar,S.,Janies,D., Hadya,N., Tang,Y., Ghedin,E. and Saif,L.
TGEV purdue	DQ811788.1	14-Jul-16	Zhang,X., Hasoksuz,M., Spiro,D., Halpin,R., Wang,S., Stollar,S.,Janies,D., Hadya,N., Tang,Y., Ghedin,E. and Saif,L.
Bat CoV isolate PREDICT/PDF 21	NC_034440.1	20-Nov-20	Anthony,S.J., Gilardi,K., Menachery,V.D., Goldstein,T., Ssebide,B., Mbabazi,R., Navarrete-Macias,I., Liang,E., Wells,H., Hicks,A.,Petrosov,A., Byarugaba,D.K., Debbink,K., Dinnon,K.H., Scobey,T.,Randell,S.H., Yount,B.L., Cranfield,M., Johnson,C.K., Baric,R.S.,Lipkin,W.I. and Mazet,J.A.
MERS-CoV	MF598663.1	15-Nov-17	Yusof,M.F., Queen,K., Eltahir,Y.M., Paden,C.R., Al Hammadi,Z.M.A.H., Tao,Y., Li,Y., Khalafalla,A.I., Shi,M., Zhang,J.,Mohamed,M.S.A.E., Abd Elaal Ahmed,M.H., Azeez,I.A., Bensalah,O.K.,Eldahab,Z.S., Al Hosani,F.I., Gerber,S.I., Hall,A.J., Tong,S. and Al Muhairi,S.S.
HKU5	EF065512.1	7-Feb-07	Woo,P.C., Wang,M., Lau,S.K., Xu,H., Poon,R.W., Guo,R., Wong,B.H., Gao,K., Tsoi,H.W., Huang,Y., Li,K.S., Lam,C.S., Chan,K.H.,Zheng,B.J. and Yuen,K.Y.
Bat CoV HKU4	NC_009019.1	20-Nov-20	Luo,C.M., Wang,N., Yang,X.L., Liu,H.Z., Zhang,W., Li,B., Hu,B.,Peng,C., Geng,Q.B., Zhu,G.J., Li,F. and Shi,Z.L.
SARS-CoV-2	NC_045512.2	18-Jul-20	Wu,F., Zhao,S., Yu,B., Chen,Y.M., Wang,W., Song,Z.G., Hu,Y.,Tao,Z.W., Tian,J.H., Pei,Y.Y., Yuan,M.L., Zhang,Y.L., Dai,F.H.,Liu,Y., Wang,Q.M., Zheng,J.J., Xu,L., Holmes,E.C. and Zhang,Y.Z.
Bat-SRBD	FJ211859.1	26-Jul-16	Becker,M.M., Graham,R.L., Donaldson,E.F., Rockx,B.R., Sims,A.C.,Sheahan,T., Pickles,R.J., Corte,D., Johnston,R.E., Baric,R.S. and Denison,M.R.
Bat SARS CoV HKU3	DQ022305.2	25-Oct-05	Lau,S.K., Woo,P.C., Li,K.S., Huang,Y., Tsoi,H.W., Wong,B.H.,Wong,S.S., Leung,S.Y., Chan,K.H. and Yuen,K.Y.
Rhinolophus affinis CoV LYRa11	KF569996.1	29-May-14	He,B., Zhang,Y., Xu,L., Yang,W., Yang,F., Feng,Y., Xia,L., Zhou,J.,Zhen,W., Feng,Y., Guo,H., Zhang,H. and Tu,C.
SARS-CoV	NC_004718	20-Nov-20	He,R., Dobie,F., Ballantine,M., Leeson,A., Li,Y., Bastien,N., Cutts,T., Andonov,A., Cao,J., Booth,T.F., Plummer,F.A., Tyler,S., Baker,L. and Li,X.
WIV1	KF367457.1	6-Nov-13	Ge,X.Y., Li,J.L., Yang,X.L., Chmura,A.A., Zhu,G., Epstein,J.H., Mazet,J.K., Hu,B., Zhang,W., Peng,C., Zhang,Y.J., Luo,C.M., Tan,B., Wang,N., Zhu,Y., Crameri,G., Zhang,S.Y., Wang,L.F., Daszak,P. and Shi,Z.L.
SHC014	KC881005.1	22-Nov-13	Ge,X.Y., Li,J.L., Yang,X.L., Chmura,A.A., Zhu,G., Epstein,J.H.,Mazet,J.K., Hu,B., Zhang,W., Peng,C., Zhang,Y.J., Luo,C.M., Tan,B.,Wang,N., Zhu,Y., Crameri,G., Zhang,S.Y., Wang,L.F., Daszak,P. and Shi,Z.L.
Rousettus bat CoV GCCDC1	NC_030886.1	3-Sep-20	Obameso,J.O., Li,H., Jia,H., Han,M., Zhu,S., Huang,C., Zhao,Y.,Zhao,M., Bai,Y., Yuan,F., Zhao,H., Peng,X., Xu,W., Tan,W., Zhao,Y.,Yuen,K.Y., Liu,W.J., Lu,L. and Gao,G.F.
Rousettus bat CoV HKU9	MG762674.1	24-Mar-18	Luo,Y., Li,B., Jiang,R.D., Hu,B.J., Luo,D.S., Zhu,G.J., Hu,B.,Liu,H.Z., Zhang,Y.Z., Yang,X.L. and Shi,Z.L.
Rabbit CoV HKU14	NC_017083.1	14-Nov-20	Lau,S.K., Woo,P.C., Yip,C.C., Fan,R.Y., Huang,Y., Wang,M., Guo,R.,Lam,C.S., Tsang,A.K., Lai,K.K., Chan,K.H., Che,X.Y., Zheng,B.J. and Yuen,K.Y.
HCoV-HKU1	KF686346.1	26-Sep-14	Dominguez,S.R., Shrivastava,S., Berglund,A., Qian,Z., Goes,L.G., Halpin,R.A., Fedorova,N., Ransier,A., Weston,P.A., Durigon,E.L., Jerez,J.A., Robinson,C.C., Town,C.D. and Holmes,K.V.

Murine CoV	KF268338.1	26-Sep-14	Town,C.D., Halpin,R.A., Ransier,A., Fedorova,N., Tsitrin,T., Mclellan,M., Stockwell,T., Amedeo,P., Bishop,B., Edworthy,P., Gupta,N., Katzel,D., Li,K., Schobel,S., Shrivastava,S., Thovarai,V., Wang,S., Siddell,S.G., Zhao,L., Elliott,R., Wentworth,D.E. and Weiss,S.R.
HCoV-OC43	KJ958219.1	19-Nov-14	Hu,Q., Lu,R. and Tan,W.
Bovine CoV	U00735.2	23-Apr-03	Hofmann,M.A., Chang,R.Y., Ku,S. and Brian,D.A.
Human enteric CoV 4408	FJ415324.1	18-Nov-08	Zhu,H., Cai,Y., Liu,Y., Yu,D., Pu,Y., Halcom,K. and Zhang,X.
Sable antelope CoV	EF424621.1	23-Jul-16	Zhang,X., Hasoksuz,M., Spiro,D., Halpin,R., Wang,S., Vlasova,A., Janies,D., Jones,L., Ghedin,E. and Saif,L.
Night heron CoV HKU19	NC_016994.1	13-Aug-18	Woo,P.C., Lau,S.K., Lam,C.S., Lau,C.C., Tsang,A.K., Lau,J.H., Bai,R., Teng,J.L., Tsang,C.C., Wang,M., Zheng,B.J., Chan,K.H. and Yuen,K.Y.
Wigeon CoV HKU20	NC_016995.1	13-Aug-18	Woo,P.C., Lau,S.K., Lam,C.S., Lau,C.C., Tsang,A.K., Lau,J.H., Bai,R., Teng,J.L., Tsang,C.C., Wang,M., Zheng,B.J., Chan,K.H. and Yuen,K.Y.
Bulbul CoV HKU11	NC_011547.1	24-Aug-18	Woo,P.C., Lau,S.K., Lam,C.S., Lai,K.K., Huang,Y., Lee,P., Luk,G.S., Dyrting,K.C., Chan,K.H. and Yuen,K.Y.
Thrush CoV HKU12	NC_011549.1	13-Aug-18	Woo,P.C., Lau,S.K., Lam,C.S., Lai,K.K., Huang,Y., Lee,P., Luk,G.S., Dyrting,K.C., Chan,K.H. and Yuen,K.Y.
Magpie robin CoV HKU18	NC_016993.1	13-Aug-18	Woo,P.C., Lau,S.K., Lam,C.S., Lau,C.C., Tsang,A.K., Lau,J.H., Bai,R., Teng,J.L., Tsang,C.C., Wang,M., Zheng,B.J., Chan,K.H. and Yuen,K.Y.
Munia CoV HKU13	NC_011550.1	13-Aug-18	Woo,P.C., Lau,S.K., Lam,C.S., Lai,K.K., Huang,Y., Lee,P., Luk,G.S., Dyrting,K.C., Chan,K.H. and Yuen,K.Y.
White eye CoV HKU16	NC_016991.1	13-Aug-18	Woo,P.C., Lau,S.K., Lam,C.S., Lau,C.C., Tsang,A.K., Lau,J.H., Bai,R., Teng,J.L., Tsang,C.C., Wang,M., Zheng,B.J., Chan,K.H. and Yuen,K.Y.
Common moorhen CoV HKU21	NC_016996.1	13-Aug-18	Woo,P.C., Lau,S.K., Lam,C.S., Lau,C.C., Tsang,A.K., Lau,J.H., Bai,R., Teng,J.L., Tsang,C.C., Wang,M., Zheng,B.J., Chan,K.H. and Yuen,K.Y.
Porcine CoV HKU15	NC_039208.1	24-Aug-18	Woo,P.C., Lau,S.K., Lam,C.S., Lau,C.C., Tsang,A.K., Lau,J.H., Bai,R., Teng,J.L., Tsang,C.C., Wang,M., Zheng,B.J., Chan,K.H. and Yuen,K.Y.
Sparrow CoV HKU17	NC_016992.1	13-Aug-18	Woo,P.C., Lau,S.K., Lam,C.S., Lau,C.C., Tsang,A.K., Lau,J.H., Bai,R., Teng,J.L., Tsang,C.C., Wang,M., Zheng,B.J., Chan,K.H. and Yuen,K.Y.
Bottlenose dolphin CoV HKU22	KF793826.1	11-Mar-14	Woo,P.C.Y., Lau,S.K.P., Lam,C.S.F., Tsang,A.K.L., Hui,S.-W., Fan,R.Y.Y., Martelli,P. and Yuen,K.-Y.
TCoV-540	EU022525.1	18-Jul-08	Cao,J., Wu,C.C. and Lin,T.L.
Avian CoV Massachusetts	AY646283.1	17-Jul-04	Fu,J.D., Liao,M., Ren,T., Zhang,G.H., Xin,C.A. and Chen,J.D.
